# *TBX5*基因在肺癌中的研究进展

**DOI:** 10.3779/j.issn.1009-3419.2020.102.27

**Published:** 2020-10-20

**Authors:** 伟嘉 黄, 培玮 李, 小明 邱

**Affiliations:** 1 600041 成都，四川大学华西临床医学院 West China School of Medicine, Sichuan University, Chengdu 610041, China; 2 610041 成都，四川大学华西医院肺癌中心 Department of Lung Cancer Center, West China Hospital, Sichuan University, Chengdu 610041, China

**Keywords:** T-box转录因子, 肺肿瘤, 基因表达, DNA甲基化, 疾病进展, T-box transcription factor gene, Lung neoplasms, Gene expression, DNA methylation, Disease progression

## Abstract

T-box转录因子（T-box transcription factor gene, *TBX*）基因涉及器官的发生，TBX5在人的正常心脏和肺组织中表达水平最高。TBX5的缺乏可能导致胸廓发育畸形和膈肌发育异常，其异位表达和过表达会诱导细胞凋亡和抑制细胞生长。既往研究发现了TBX5在食管腺癌、胃癌、结肠癌和乳腺癌的发生和发展中的潜在作用。我们对TBX2亚家族的基因表达和预后之间的关系进行了综述，同时探究TBX5在调控肺癌发生发展机制中的研究进展。虽然TBX5和肺癌发生之间的关系尚不明确，不过TBX5可以显著抑制人体内肿瘤生长，其表达水平和肺癌的进展呈现负相关。由此，TBX5的基因表达水平和甲基化程度是潜在的表证肺癌增殖和转移的生物标志物，具有作为肺癌治疗靶点的潜力。

## Abstract (en)

T-box transcription factor gene (*TBX*) interferes with the origin and development of organs, and TBX5 is expressed highest in normal cardiac and pulmonary tissues. Lack of TBX5 may lead to thoracic malformation and abnormal diaphragmatic development, in which ectopic expression and overexpression may induce the apoptosis of cell and inhibit the development of cell. Previous studies demonstrated the potential role of TBX5 in the development and progression of esophageal adenocarcinoma, gastric cancer, colon cancer and breast cancer. We reviewed the association between the expression of TBX2 subfamily and the prognosis, and explore the research progress of TBX5 in regulating the development and progression of lung cancer. Even though the relationshihp the development of lung cancer and TBX5 are not clear, TBX5 could significantly inhibit in vivo tumor growth, and the level of TBX5 was negatively correlated with lung cancer progression. Therefore, the gene expression levels and methylation extent of *TBX* could be a potential biomarker to reveal the proliferation and metastasis of lung cancer, as well as a therapeutic target for lung cancer.

## 1 TBX5的概述

T-box转录因子（T-box transcription factor gene, *TBX*）基因拥有一个共同的DNA结构域T-box，作为一种高效的DNA结合转录激活因子可编码一系列调节进化保守的涉及器官发生和发育的转录因子，并且相同基因的不同选择性剪切亚型在细胞增殖中起到不同作用^[1]^。TBX家族的基因突变可能导致发育综合征，而目前越来越多的研究支持这些变化在某些癌症中发挥一定作用^[2]^。以往对于TBX家族基因的研究仅涉及器官发育^[2-5]^，但是目前已有少量学者开始研究TBX家族的基因在正常和癌症组织中的表达水平^[6, 7]^，并由此探究其作为生物标志物在肺癌发生和转移机制中的作用。

TBX2亚家族（TBX2-5）在人体组织中均有表达，TBX5在心脏、肺、前肢、食管、膀胱、乳腺、前列腺和甲状腺等组织中均有表达，而TBX5在正常心脏和肺组织中表达水平最高^[8]^。TBX5在肺芽的发展中有直接的作用，TBX4和TBX5通过成纤维细胞生长因子10（fibroblast growth factor 10, FGF10）信号通路的激活共同调控肺分支的形态发生^[3]^。TBX5的异位表达和过表达会抑制集落形成、诱导细胞凋亡和抑制细胞生长，在正常细胞中的表达可使发育受阻，在癌细胞中的表达可抑制癌细胞的增殖和迁移^[9, 10]^。

目前，TBX5被认为参与到了食管腺癌和结肠癌的发生过程^[11, 12]^。Palles等^[13]^通过全基因组关联研究发现*TBX5*（rs2701108）与Barrett食管和食管腺癌的发生风险相关。而与编码Barrett食管转录因子的基因也参与到了胸廓、膈肌和食管的发育中。Becker等^[11]^的研究同样证实了rs2701108在Barrett食管和食管腺癌发生中的作用，推测TBX5在膈肌的组织的发育过程中起重要作用，而*TBX5*基因突变可能导致食管裂孔疝进而导致胃食管反流病，最终导致Barrett食管进展为食管腺癌。此外，β-catenin-TBX5复合体（Yes-associated protein 1, YAP1）在β连环蛋白（β-catenin）驱动的癌症，特别是结肠癌的发生发展中具有重要作用^[12]^。

启动子甲基化被认为是抑制肿瘤相关基因尤其是抑癌基因失活的主要机制之一，伴随着基因的改变，最终导致癌症的发生^[14-17]^。*TBX5*甲基化可在68%的原发性结肠癌中检测到，同时与总生存不佳显著相关（*P*=0.000, 7）。TBX5在结肠癌细胞中的表达水平很低，甚至不表达，而TBX5的再表达可抑制癌细胞增殖（*P* < 0.001）和诱导癌细胞凋亡（*P* < 0.01）。TBX5通过上调细胞周期蛋白依赖的激酶抑制剂2A（cyclin-dependent kinase inhibitor 2A, CDKN2A）和转移抑制因子1（metastasis suppressor 1, MTSS1），下调γ-神经突触核蛋白（synuclein gamma, SNCG）、转移相关基因2（metastasis-associated 1 family member 2, MTA2）来抑制肿瘤细胞增殖和转移。TBX5通过外源性凋亡途径与B淋巴瘤-2基因相关X蛋白（BCL2-associated X protein, BAX）和颗粒酶A的表达水平诱导结肠癌肿瘤细胞凋亡^[10]^。因此，*TBX5*甲基化程度可能是反映结肠癌患者生存的预测因子。

此外，微小RNA-10b（microRNA-10b, miR-10b）的上调与肿瘤的侵袭性和转移性密切相关^[18, 19]^。miR-10b通过抑制转录因子TBX5的表达，进而抑制抑癌基因双特异性酪氨酸磷酸化调节激酶1A基因（gene of dual specificity tyrosine phosphorylation regulated kinase 1A, *DYRK1A*）和与张力蛋白同源在10号染色体有缺失的磷酸酶基因（gene of phosphate and tension homology deleted on chromsome ten, *PTEN*）的表达，最终促进了乳腺癌细胞增殖、迁移和侵袭。而临床结果^[20]^也证实，相较于TBX5高表达的乳腺肿瘤患者而言，TBX5低表达患者的无复发生存和总生存更差。不过在Ⅰ期和Ⅱ期的胃癌患者中，Zheng等^[7]^发现高表达的TBX5反而与不良生存相关，而Ⅲ期和Ⅳ期胃癌患者，肿瘤组织中的TBX5表达水平与正常组织并没有显著差异。由此，TBX5在肿瘤组织中的表达水平可能是潜在的预后指标。

## 2 TBX5在肺癌中的表达

目前，不少研究^[21]^认为TBX5和肺癌发生的关系尚不明确。Khalil等^[8]^的研究发现相较于正常肺组织，TBX2亚家族在非小细胞肺癌中的表达受到显著抑制（*P* < 1×10^-9^），在肺癌前病变组织中TBX2亚家族基因水平均有所下降（*P* < 1×10^-4^），所以TBX2亚家族基因水平的下调可能与早期非小细胞肺癌的发生相关。该研究结果与另一项研究体外实验^[22]^的结果一致。

Arora等^[3]^的研究发现TBX4和TBX5在生长中小鼠的肺和气管的间充质中均有表达，但在上皮细胞中不表达，TBX5的缺失可仅使单肺发育而气管并不形成，TBX4的缺失则并不会有这样的影响。TBX4和TBX5均通过FGF10来调节肺分支的形成，通过性别决定区域Y盒（sex-determining region Y-box, *SOX9*）基因以独立于独立于FGF10的途径控制气管或支气管软骨形成。Horie等^[23]^发现TBX4参与到关键成纤维细胞作用相关的基因转录调节，也细胞中和超增强子相关的基因转录调节。肺癌相关成纤维细胞可能是由常驻成纤维细胞发展而来，而肺特异性成纤维细胞特征的丧失可能是其表型转化为癌症相关成纤维细胞的原因。病理情况下，肺成纤维细胞中表达较高的基因，尤其是转录因子，在肺癌相关成纤维细胞中均被抑制，而TBX2、TBX4和TBX5的沉默与肺癌相关成纤维细胞中的DNA高甲基化有关。

早先有研究提出癌症域（field of cancerization）^[24]^和组织损伤域（field of tissue injury）^[25]^，而相较于无瘤的吸烟者，患有肺癌的吸烟者在正常支气管组织中的TBX2亚家族基因表达减少，同时具有可疑结节的吸烟者的支气管组织中TBX2亚家族基因表达显著减少（*P* < 1×10^-5^）。所以TBX2亚家族的表达受抑制可能在非小细胞肺癌的发病早期发生，其基因表达水平可以作为具有可疑结节的高风险吸烟者检测肺癌的生物标志物^[8]^。Ma等^[26]^在他们的研究中，首次提出TBX5在非小细胞肺癌的正常邻近组织中高度表达，但是在非小细胞肺癌的组织和细胞中，其水平显著下调。因此表明TBX5的水平和肺癌的进展呈负相关。基于组织微阵列的免疫组化染色结果，他们发现非小细胞肺癌组织内TBX5表达水平和肿瘤原发灶-淋巴结-转移（tumor-node-metastasis, TNM）分期（*P*= 0.016）、组织病理学类型（*P*=0.029）和淋巴结状态显著相关（*P*=0.035），而与年龄、性别和病理学分化没有关系（*P* > 0.05）。在裸鼠中的实验发现TBX5水平增加显著减弱了细胞的致瘤能力，TBX5过表达使肿瘤体积和质量显著降低，由此证明了TBX5可以在体内发挥肿瘤抑制作用。不过相反的是，相较于邻近正常组织，同属于TBX2亚家族的TBX2在非小细胞肺癌组织中的表达水平显著升高（*P* < 0.001），同时其表达水平和组织学类型（*P*=0.014）、淋巴结转移情况（*P*=0.008）和远处转移情况（*P*=0.021）显著相关。TBX2的表达水平对于总生存期来说也是独立的预后指标（HR=2.21, *P*=0.001, 5），反映了癌组织的转移潜力^[27, 28]^。Yu等^[29]^的研究进一步指出TBX2可能是晚期肺腺癌骨转移的潜在指标。所以TBX2亚家族不仅可能作为肺癌的治疗靶点，其基因表达水平也是潜在的诊断肺癌和判断预后的生物标志物。

除了TBX2，TBX3-5的mRNA表达水平均和基因的甲基化程度呈负相关（*P* < 0.05）^[17]^。Shi等^[30]^在全基因组范围内对鳞状细胞肺癌的表观遗传模式进行了研究，通过微阵列研究并结合DNA甲基化和mRNA表达数据鉴定出449个伴随表达改变的异常甲基化基因，在经过基因本体论（gene ontology, GO）分析和焦磷酸测序后鉴定出*CLDN1*、*TP63*、*TBX5*、*TCF21*、*ADHFE1*和*HNF1B*等6个DNA甲基化生物标志物。受试者工作特征（receiver operating characteristics, ROC）曲线分析纳入了癌症基因组图谱（The Cancer Genome Atlas, TCGA）数据库的数据进行验证，结果显示曲线下面积（area under the curve, AUC）为0.984，所以TBX5可能是潜在的诊断肺鳞癌的生物标志物。虽然在肺鳞癌和肺腺癌中TBX2-5的甲基化程度均显著升高，特别是TBX4和TBX5，不过分期（Ⅰ期-Ⅱ期*vs* Ⅲ期-Ⅳ期）和吸烟史似乎对甲基化程度没有显著影响。由此，TBX2亚家族的甲基化可能发生在非小细胞肺癌的发生早期^[17]^。Nawaz等^[31]^的研究发现多重甲基化特异性PCR（multiplex methylation specific polymerase chain reaction, MMSP）可用于检测非小细胞肺癌的转移潜力，他们发现有两种组合的灵敏度和特异性最高，均为86%和97%，分别是HOXA9、TBX5、PITX2和HOXA9、TBX5、PITX2、RASSF1A。不过他们的研究中提到这种监测仅可用于治疗后的随访和癌症的转移风险。

## 3 TBX5调控肺癌发生发展的机制研究

TBX5在发育的心脏和肢体芽中呈现梯度分布，表明TBX5在体内具有不同的作用^[32, 33]^。TBX5过表达显著抑制体外非小细胞肺癌细胞的增殖、集落形成和侵袭并诱导细胞凋亡。Ma等^[26]^首次提出TBX5水平与人肺癌进展呈负相关，在裸鼠内TBX5水平升高可基本阻断肿瘤的生长，所以TBX5可以作为非小细胞肺癌的诊断和治疗的靶标。而Bruneau等^[34]^的研究提出TBX5存在于成熟心脏中和心脏的肿瘤发生率低可能存在一定关系，可能是TBX5潜在地抑制某些肿瘤细胞的生长，并诱导细胞凋亡^[9]^。

而Khalil等^[22]^进一步提出TBX2-5的下调并不是TBX阴性表达的细胞扩增所致，而是由于细胞的自主调节机制所致，这对于其能否作为可靠的生物标志物极为关键。他们的研究提出TBX2-5共同构成了抑制肺癌进展的通路^[22]^，这也被Ma等^[26]^包括非小细胞肺癌在内的不同癌细胞系的研究所证明。TBX2-5能够通过调节甲基化和去甲基化过程来抑制细胞周期进程，而甲基化和去甲基化过程可影响与癌症发展和进展有关的下游基因的表达^[1, 22]^。

不过Khalil等^[22]^发现在他们的研究之前并没有学者提出TBX4-5在其他癌细胞系中过表达，而有研究认为TBX2和TBX3可以通过靶向CDKN1A和CDKN1B^[35]^以及*CDKN2A*基因特异性影响衰老^[36]^，但是Khalil的研究认为*CDKN2A*基因的激活程度轻，与其在细胞增殖中起到的主要抑制作用不符，并不能特异性影响衰老^[22]^。同时，Khalil等^[22]^提出他们实验中所采用的转录组测序（RNA-seq）技术是第一个在癌症环境中无偏移评价TBX2-5作用的方法，而与其他研究所采用的“先验”方法不同。He等^[9]^的实验发现TBX5可凭借不同的敏感性有效抑制不同癌细胞的集落形成，包括U2OS细胞、HeLa细胞、H1299细胞和Saos-2细胞，而TBX5的异位表达会导致细胞凋亡并抑制细胞生长。

Qiao等^[37]^分析了来自GEO数据库的非吸烟女性肺癌的微阵列数据，发现TBX5-AS1（TBX5 antisense RNA 1）等3个长链非编码RNA（long non-coding RNA, lncRNA）在肺癌组织中显著下调，而另有5个lncRNA显著上调。Xiong等^[38]^的研究发现TBX5-AS1的高表达也与肺鳞癌的不良预后相关（*P*=0.023）。AFAP1-AS1的上调与非小细胞肺癌患者的预后不良相关^[39]^，其他的研究^[40-44]^也提出肺癌组织中lncRNA表达水平的上调与肺癌的增殖、侵袭和转移相关。目前，TBX5-AS1的生物学功能并不清楚，但是Qiao等^[37]^推测其异常表达可能在女性肺癌的发生发展中有一定作用。

有研究^[45]^认为沙利度胺可以抑制细胞增殖和诱导细胞凋亡，同时可以特异性地和TBX5结合。不过，研究^[22, 45-47]^认为Khalil的体外实验和有关沙利度胺的临床试验都是失败的，没有对肿瘤细胞有很好的抑制作用。Jazieh等^[46]^的Ⅱ期单臂临床试验的中位总生存期为10.8个月，中位无进展生存期为4个月。Hoang等^[48]^有关沙利度胺的Ⅲ期临床试验（ECOG 3598）发现放化疗联合沙利度胺并未改善局部晚期非小细胞肺癌患者的总生存期，与对照组相比，3级不良反应的发生率更高（53% *vs* 44%, *P*=0.004）。Nemer等^[45]^的实验并未证明在使用沙利度胺后，细胞因子和黏附分子发生变化，沙利度胺的抑癌作用可能对于肺腺癌并不适用。

## 4 结论

目前对于TBX2亚家族的研究已逐渐从器官的发生发育转向肿瘤的发生、增殖和转移，而TBX5已经被发现和肺癌、食管癌、胃癌、乳腺癌和结肠癌的发生与转移之间存在一定关系。目前的研究对于TBX5与肺癌发生的机制并没有很好的阐述，不过，已经有研究提出包括*TBX5*在内的四种TBX2亚家族基因在NSCLC中表达受抑制。在癌前病变的组织中，其表达水平相对于正常组织也有所下降，而TBX2-5可以独立调控肺癌的进展。动物实验和表观遗传学等相关研究提出了基因异常甲基化的现象，TBX5会因启动子被甲基化而沉默或降低活性，因而无法抑制肿瘤细胞的恶性增殖。因此，调节TBX5的表达水平可能是调控肺癌发生和进展的途径，TBX5的表达水平和甲基化程度是潜在的用以诊断肺癌和判断预后的生物标志物，不过需要进一步的实验阐明TBX5通路的机制和作用，同样也需要更多的临床样本阐述TBX5在肺癌中的表达及其预后价值。
